# Use of Contaminated Habitat and Associated Selenium Uptake Mediate Haemosporidian Parasite Infections in Wild Passerine Birds

**DOI:** 10.1002/ece3.72681

**Published:** 2026-01-13

**Authors:** Courtney S. Werner, Mary Chapman, Daniel A. H. Peach, Travis L. DeVault, Olin E. Rhodes

**Affiliations:** ^1^ Department of Fish and Wildlife Conservation Virginia Polytechnic Institute and State University Blacksburg Virginia USA; ^2^ Savannah River Ecology Laboratory University of Georgia Aiken South Carolina USA; ^3^ Department of Infectious Diseases, College of Veterinary Medicine University of Georgia Athens Georgia USA; ^4^ Warnell School of Forestry and Natural Resources University of Georgia Athens Georgia USA; ^5^ Odum School of Ecology University of Georgia Athens Georgia USA

**Keywords:** avian, coal combustion, disease, heavy metal, malaria, metalloid, radionuclide

## Abstract

Environmental contamination alters ecological interactions among organisms, including those associated with parasitism. Contaminants can mediate parasitic relationships at multiple scales by changing host vulnerability to infection and disrupting transmission‐relevant contacts. The overall effect of contamination on parasitism remains poorly understood, yet the interplay between these stressors has significant implications for animal and human health. We conducted a community‐scale field study to evaluate whether trace element contaminants derived from coal combustion residuals and nuclear fission products alter the dynamics of haemosporidian blood parasites, dipteran vectors, and avian hosts in riparian and wetland habitats in South Carolina, USA. We captured 329 individuals of 31 passerine bird species and 195 *Culex* mosquito vectors at two sites affected by coal combustion waste, two sites affected by nuclear fission waste, and two reference sites. We evaluated whether blood concentrations of zinc, copper, mercury, and selenium and whole‐body radioactivity concentrations because of cesium‐137 predicted the likelihood of single and coinfections by *Plasmodium*, *Haemoproteus*, and *Leucocytozoon* within passerine hosts. We also evaluated whether the likelihood of *Plasmodium* infection in *Culex* vectors differed with the presence of site‐level contamination. Individual passerine hosts inhabiting coal combustion waste sites had significantly higher blood selenium concentrations than those at reference sites, and blood selenium was negatively associated with the likelihood of *Leucocytozoon* infection. The likelihood of infection with *Plasmodium* did not vary between vectors at contaminated versus reference sites. The transfer of low‐dose, waste‐derived selenium to wildlife may bolster individual response to some parasites and increase the reservoir capacity of host populations. Our findings highlight complex effects of trace elements on wildlife disease dynamics and reveal priorities for future research in contaminated habitat.

## Graphical abstract

We conducted a community‐scale field study to evaluate whether trace element contaminants derived from coal combustion residuals and nuclear fission products alter the dynamics of haemosporidian blood parasites, dipteran vectors, and avian hosts in riparian and wetland habitats in South Carolina, USA. Individual passerine hosts inhabiting coal combustion waste sites had significantly higher blood selenium concentrations than those at reference sites, and blood selenium was negatively associated with the likelihood of *Leucocytozoon* infection. The transfer of low‐dose, waste‐derived selenium to wildlife may bolster individual response to some parasites and increase the reservoir capacity of host populations.

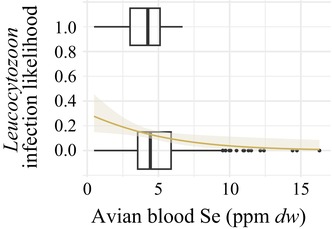

## 1 Introduction

Anthropogenic waste fundamentally alters the chemistry of Earth's ecosystems (UNEP 2020). Processes associated with mining, coal combustion, hydraulic fracturing, and nuclear fission generate byproducts that contain metals, metalloids, and radionuclides, many of which occur naturally in trace amounts but are released to the environment in excess concentrations or new forms (Deonarine et al. 2023; Dwivedi et al. 2022; Estrada and Bhamidimarri 2016). Living organisms evolved in the presence of naturally occurring trace elements and possess adaptive mechanisms for processing them, but influxes of trace element waste rapidly transform ecosystem properties and create novel chemical environments in which organisms interact (Kapustka 2004). Chemical stressors alter the movement, nutrition, reproduction, and immune function of individuals and exert selective pressures on populations, with consequences for ecosystem function and human and animal health (Kramer et al. 2010; Saaristo et al. 2018).

Parasites, vectors, and hosts maintain dynamic ecological and evolutionary relationships that are affected by disturbances to their shared environments (Morales‐Castilla et al. 2021; Patz et al. 2000; Werner and Nunn 2020). Trace metal or radionuclide contamination can alter these relationships at multiple scales, with multifaceted effects on parasite transmission (Lafferty and Kuris 1999; Marcogliese 2005). One possible outcome of such alterations is greater parasitism among hosts inhabiting contaminated environments. For instance, exposure to excess trace elements or ionizing radiation can induce oxidative stress and immunosuppression (Becker et al. 2017; Kesäniemi et al. 2019; Koller 1980), thereby weakening hosts' adaptive defenses against parasite infection (Morley et al. 2006). Immunosuppression may allow a greater number of parasite species to establish infections in a host, with consequences for disease severity (Pigeault et al. 2018). Contaminants can also impair host movement (Kojima et al. 2024) and limit behavioral defenses such as grooming (Burbacher et al. 1990), resulting in higher rates of exposure to vector‐transmitted parasites. At a landscape scale, environmental contamination can degrade habitat quality and reduce food availability for hosts, leading to indirect effects of contaminants on host nutrition, body condition, and immune function (Sánchez et al. 2020). The improper disposal of trace element waste and subsequent contamination of natural resources can exacerbate the severity of disease impacts on vulnerable communities of wildlife, domestic animals, and humans (Cable et al. 2022; Krystosik et al. 2020).

Contaminants can also decrease parasitism among hosts in polluted habitats, either through direct toxicity to parasites or by disrupting transmission (Sures et al. 2017). Heteroxenous parasites rely on the integrity of multiple ecological interactions between their definitive and intermediate hosts or vectors to complete their development and are estimated to be in decline globally because of biodiversity loss (Dunn et al. 2009; but see Keesing and Ostfeld 2021). The presence or richness of heteroxenous parasites has been proposed as a metric of ecosystem integrity, with diminished parasite communities indicating impaired ecosystem function in disturbed environments (Marcogliese 2005; Sures et al. 2017). Contaminant‐induced changes to host or vector fitness, population size, and movement patterns can suppress parasites if the pathways integral to their transmission are disrupted (Neff and Dharmarajan 2021; Sánchez et al. 2020). Many heteroxenous parasites of terrestrial vertebrates undergo partial development within hosts or vectors that have aquatic life stages and are particularly vulnerable to the accumulation of trace element contaminants in freshwater systems (Marcogliese 2005; Poulin 1992). Additionally, if definitive hosts experience rapid mortality after contaminant exposure, then host density and transmission‐relevant contact rates are likely to decline (Sánchez et al. 2020). The study of heteroxenous parasites in contaminated ecosystems can alert investigators to the loss of their hosts or vectors and serve as an indication of community‐scale toxicity impacts (Sures et al. 2017).

Studies investigating the relationships between contaminants, parasites, and hosts have revealed a range of effects, likely because of the contradictory factors that govern these complex relationships in different systems. Prior work has occurred primarily in freshwater environments and has demonstrated that parasitism can increase or decrease in aquatic wildlife exposed to trace element pollution, with the direction and magnitude of effects dependent on system‐specific factors like parasite life cycle and contaminant bioavailability (Poulin 1992; Sures et al. 2017). Relatively fewer studies have investigated the effects of trace element pollution on parasitism in terrestrial wildlife (Cable et al. 2022; Riley et al. 2014), and even fewer have incorporated vectors or intermediate hosts when examining how contaminants mediate the host–parasite relationship (Krystosik et al. 2020). Despite this, terrestrial wildlife are known to accumulate contaminants in habitats affected by anthropogenic waste (Ackerman et al. 2016; Becker et al. 2018; Oldenkamp et al. 2017) and serve as primary reservoirs of zoonotic parasites with paramount medical and veterinary importance (Daszak et al. 2000; Miller et al. 2013). Furthermore, the precipitous declines of several terrestrial wildlife species are attributed to either contaminant toxicity (Bowerman et al. 1995; Finkelstein et al. 2012) or disease (Atkinson and Samuel 2010; Hoyt et al. 2021), and a greater understanding of the interactions between these stressors is necessary for proper management of threatened populations (Riley et al. 2014).

Passeriformes are the most speciose and widespread of the avian orders and are often abundant in disturbed environments (Maklakov et al. 2011; Oliveros et al. 2019; Seewagen and Newhouse 2018). Passerines accumulate heavy metals, metalloids, and radionuclides through trophic transfer and contact with contaminated substrates (Ackerman et al. 2016; Sternalski et al. 2015; Werner et al. 2024) and have long served as bioindicators of environmental pollution (Burger 1994; Krivolutski et al. 1999). Passerines also harbor parasites that have significantly impacted naïve bird species, poultry, and humans (Atkinson and Samuel 2010; Ayala et al. 2020; Ezenwa et al. 2006). Perhaps the best‐studied avian parasites are haemosporidia of the genus *Plasmodium*, which are closely related to the agents of human malaria and are primarily vectored by *Culex* mosquitoes (Valkiunas 2004). *Plasmodium* in wild birds often co‐occur with the haemosporidia *Haemoproteus* and *Leucocytozoon*, which are vectored by biting midges (*Culicoides*) and simuliid flies (Simuliidae), respectively (Valkiunas 2004). Molecular evidence indicates that haemosporidia originated in palaeognathous birds approximately 68 Ma, prior to the superradiation of Passeriformes (Oliveros et al. 2019; Pacheco et al. 2018). This extensive coevolutionary history is evident in the ubiquity of haemosporidian parasites among global passerines and the high host specificity of some lineages (Lotta et al. 2019; Pacheco et al. 2018). Haemosporidia infect the erythrocytes of their hosts and cause a range of deleterious effects, including anemia, oxidative stress, reduced reproductive success, and mortality (Dadam et al. 2019; Pigeault et al. 2018; Valkiunas 2004). Disease severity varies with the presence of coinfecting parasites or other stressors and can drive population declines, especially among naïve populations (Atkinson and Samuel 2010; Meister et al. 2021) or those affected by multiple anthropogenic disturbances (Bichet et al. 2013; Dadam et al. 2019).

Avian haemosporidia are widely used as models for understanding the eco‐evolutionary interactions between hosts, vectors, and parasites (Ellis et al. 2020; Lotta et al. 2019; Pigeault et al. 2018) and are therefore excellent candidates for evaluating how environmental contamination mediates these relationships. This study investigated whether blood concentrations of four heavy metals and two metalloids—zinc (Zn), copper (Cu), lead (Pb), mercury (Hg), selenium (Se), and arsenic (As)—and whole‐body radioactivity concentrations because of the radionuclide cesium‐137 (^137^Cs) predicted the likelihood of single and coinfections by *Plasmodium*, *Haemoproteus*, and *Leucocytozoon* within passerine hosts inhabiting two sites affected by coal combustion waste, two sites affected by nuclear fission waste, and two reference sites. We also evaluated whether the likelihood of *Plasmodium* infection in *Culex* vectors differed with the presence of site‐level contamination. We tested two hypotheses that represent the potential, divergent effects of contaminants on vector‐transmitted parasites at different ecological scales: (1) sublethal contaminant exposure increases parasitism by fostering favorable conditions for parasite communities within hosts, and (2) environmental contamination decreases parasitism by disrupting the transmission of parasites among vector communities. Evidence in support of the first hypothesis would include a higher likelihood of haemosporidian single and/or coinfection in passerine hosts with higher metal, metalloid, or radionuclide burdens, whereas evidence in support of the second hypothesis would include a lower likelihood of haemosporidian infection in *Culex* vectors at contaminated sites compared to reference sites and in hosts that are year‐round residents of contaminated areas compared to those that are recent migrants. We aim to provide a nuanced perspective on how altered environmental chemistry because of industrial waste affects interactions between parasites, vectors, and terrestrial wildlife.

## 2 Materials and Methods

### 2.1 Site Description

The Savannah River Site (SRS) is a U.S. Department of Energy facility in the upper coastal plain of South Carolina that historically produced materials for national defense. Ten percent of the 780 km^2^ property is characterized by industrial use, and the remaining area contains managed forests and wetlands (SRNS 2022). Habitat located upstream of industrial facilities is minimally impacted by site operations, but areas adjacent to and downstream of former nuclear reactors and coal‐fired power plants are impacted by legacy wastes from releases of nuclear fission products (NFP) and coal combustion residuals (CCR; Carlton et al. 1992). Ecosystems on the SRS are therefore exposed to varying concentrations of waste‐derived trace elements and radionuclides, providing unique opportunities to assess the ecological effects of these contaminants in field settings.

### 2.2 Avian Sampling

We captured passerines and *Culex* spp. at six sites on the SRS within riparian and wetland habitats. Craig's Pond (reference 1) and Upper Three Runs (reference 2) are located upstream of point‐source contamination and have been preserved as ecological reference areas since 1952 (White and Gaines 2000). D‐Area (CCR 1) and McQueen's Branch (CCR 2) are located within the same drainage basins as coal combustion waste areas that serve as ongoing sources of metals and metalloids to the surrounding ecosystems (SRNS 2020). R‐Canal (NFP 1) and Steel Creek (NFP 2) are freshwater systems that received reactor cooling effluent contaminated with NFP and are primarily affected by the radionuclide ^137^Cs (Carlton et al. 1992). Additionally, the reactor cooling water contained high levels of Hg because of waste outflow from a chlor‐alkali plant located upstream on the Savannah River (White and Gaines 2000).

We used mist nets to capture passerines during the breeding season from March to July of 2023. We spent two weeks at each site before rotating, ensuring that CCR, NFP, and reference site types were sampled throughout the breeding season. Each of the four reference and CCR sites was visited twice, for a total of four weeks per site. Because of equipment constraints, NFP sites were each visited once, for a total of two weeks per site. At each site, we placed four to ten 30 mm mist nets at the edges of wetlands and streams and in adjacent forested habitat. We opened mist nets from sunrise to approximately 1100 and checked them every 20 min. We aimed for a sample that represented the full community of resident and migrant passerines and used song callbacks in an effort to obtain an even number of species across site types.

We measured the body mass of each bird and attached an aluminum USGS band at the time of capture. We did not record demographic data for the majority of individuals, nor did we take morphological measurements beyond body mass. We then collected 0.07–0.30 mL of blood from the jugular vein using an insulin syringe fitted with a 28‐gauge needle. For parasite diagnostics, three to four drops of blood were stored on Whatman FTA cards (Qiagen), chilled in the field, and transferred to a refrigerator in the lab on the day of capture. The remaining blood was transferred to metal‐free vials for trace element analysis.

We quantified whole‐body radioactivity concentrations because of ^137^Cs (Bq/g wet weight) and blood concentrations of Zn, Cu, Pb, As, Se, and Hg (ppm dry weight) according to methods detailed in Werner et al. (2025). Briefly, we measured the gamma radiation emitted from each live bird for 30 min using a high‐purity germanium (HPGe) spectrometer (Aegis; Mirion Technologies) attached to a robust Pb shield and mounted within a trailer at our field site. We used the Genie 2000 spectroscopy software to locate peaks in the ^137^Cs region of interest (662 keV) that were significantly above the Critical Level (Lc; *α* = 0.05) calculated according to Currie (1968). If ^137^Cs was present, its radioactivity concentration in the sample was determined after subtracting background radiation derived from field blanks and applying an efficiency correction tailored to the geometry of our counting system. When radioactivity due to ^137^Cs was below the critical level, ^137^Cs was assumed to be absent, and a value of zero was used for that sample (USEPA 1991). In the laboratory, we analyzed blood samples for Zn, Cu, Pb, As, and Se using an Inductively‐Coupled Plasma Mass Spectrometer NexION 300X (PerkinElmer) and for Hg using a Direct Mercury Analyzer (DMA‐80, Milestone Shelton, CT, USA). Reference materials (TORT‐3 and DOLT‐5) and a blank were included in each set of samples, and method detection limits (MDL) were calculated for each element. When a trace element was undetectable in a blood sample, a value equivalent to one half of the instrument method detection limit (MDL) was assigned for that sample (USEPA 1991).

### 2.3 Vector Sampling

Once every four weeks from April to July, we captured *Culex* spp. at all six sites simultaneously using infusion‐based gravid traps designed to attract ovipositing adult female *Culex* mosquitoes (Williams and Gingrich 2007). To mirror locally available oviposition substrates, we prepared an oak leaf infusion by steeping 1 kg of dried oak leaves obtained from the SRS in 30 L of well water for eight days (Allan et al. 2005; O'Meara et al. 1989). Frommer Updraft Gravid Traps (Model 1719, John Hock Company) were filled with five liters of infusion, placed in the field between 1300 and 1800 h, and picked up 10–16 h later. Traps containing live mosquitoes were transferred to the laboratory immediately and placed in a −20°C freezer to euthanize mosquitoes.

After euthanasia, we identified gravid *Culex* females using a dissection microscope and a morphological identification key (Burkett‐Cadena 2013). Each *Culex* female was bisected at the junction of the thorax and abdomen using sterilized forceps and a scalpel (Foley et al. 2012), and cephalothoraxes were stored at −80°C in 95% ethanol until DNA extraction.

### 2.4 Molecular Analysis

Parasite DNA was extracted from one 3‐mm hole punch of avian dried blood per bird and from the cephalothoraxes of *Culex* using the DNeasy Blood and Tissue Extraction Kit (Qiagen). We evaluated the presence of *Plasmodium*, *Haemoproteus*, and *Leucocytozoon* in each avian blood sample using a multiplex PCR assay with the primer sets PMF/PMR, HMF/HMR, and LMF/LMR (Ciloglu et al. 2019). The one‐step multiplex PCR amplifies regions of mtDNA specific to each parasite genus and does not result in non‐specific amplification, thereby providing a cost‐effective alternative to Sanger sequencing for genus‐level identification (Ciloglu et al. 2019). Each reaction contained 5 μL of commercial master mix (2× Qiagen Multiplex PCR Master Mix, Qiagen, Hilden, Germany), 0.2 μL of each of the six primers (10 μM), 1.8 μL of nanopure H_2_O, and 2 μL of template DNA. An initial denaturation step of 95°C for 15 min was followed by 35 cycles of 94°C for 30s, 59°C for 90s, and 72°C for 30s, with a final annealing step at 72°C for 10 min. Positive controls derived from a triple‐infected *Quiscalus quiscalus* and verified by microscopy were included in every PCR run along with negative controls (H_2_O). Amplification products were visualized on 2% agarose gels containing GelRedTM gel stain (Biotium Inc., Hayward, CA, USA) in a Gel Doc XR+ with Image Lab Software (Bio‐Rad, CA, USA). We noted the presence of bands corresponding to *Plasmodium* (378 bp), *Haemoproteus* (533 bp), and/or *Leucocytozoon* (218 bp) infection in each sample.

We evaluated the presence of *Plasmodium* in each *Culex* cephalothorax sample using a nested PCR with the primer pairs HaemNF1/HaemF and HaemNR3/HaemR2 to amplify a 480 bp fragment of the *cytb* gene (Hellgren et al. 2004). Each 10 μL reaction mix contained 5 μL of commercial master mix (2× Qiagen Multiplex PCR Master Mix, Qiagen, Hilden, Germany), 3.1 μL of nanopure H_2_O, 0.5 μL MgCl_2_, 0.2 μL of each primer (10 μM), and 1 μL of template DNA (25 ng/μL). The first reaction used the primer pairs HaemNF1 and HaemF and was carried out with an initial denaturation step of 95°C for 15 min followed by 20 cycles of 94°C for 30s, 50°C for 30s, and 72°C for 45 s, with a final annealing step at 72°C for 10 min. The second reaction used 1 μL of PCR product as template DNA and the primers HaemF and HaemR2. The cycling parameters were the same as the initial reaction, but with 35 cycles. Positive and negative controls (H_2_O) were included in every PCR run. We visualized amplification products on 2% agarose gels containing GelRedTM gel stain (Biotium Inc., Hayward, CA, USA) in a Gel Doc XR+ with Image Lab Software (Bio‐Rad, CA, USA) to detect the presence of a band corresponding to *Plasmodium*.

### 2.5 Data Analysis

All statistical analyses were conducted in R 4.5.1. We used phylogenetic generalized linear mixed effects models (PGLMMs) to evaluate whether concentrations of Zn, Cu, Pb, Se, As, Hg, and ^137^Cs in birds varied with site contaminant history, species relatedness, or species identity independent of relatedness. We obtained a distribution of 1000 avian phylogenetic trees from BirdTree.org (Ericson et al. 2006; Jetz et al. 2012) and used *phytools::minTreeDist* to identify the single tree topology with branch lengths that minimized distance to all other trees (Revell 2024). For each contaminant, we used *phyr* to fit a PGLMM that included a fixed effect for site contaminant history (CCR, NFP, or reference) and random effects for both phylogenetic covariance and independent species variance (Li et al. 2020). For contaminants detected in fewer than half of the bird samples (Pb, As, and ^137^Cs), we used a binary response variable indicating whether the contaminant was detected and fit a binomial family PGLMM with a logit link. For all other contaminants, we fit a Gaussian family PGLMM with an identity link.

We used a second set of PGLMMs to evaluate whether haemosporidian infection in birds was predicted by (1) blood concentrations (ppm *dw*) of metal(loid) contaminants in individuals, (2) whole‐body radioactivity concentrations (Bq/g *ww*) of ^137^Cs, and (3) month of capture. The concentrations of metals in the blood and the activity concentrations of ^137^Cs in individual birds were included as fixed effects after each was standardized to have a mean of zero and standard deviation of one. We evaluated concentrations for multicollinearity by evaluating whether Pearson's correlation coefficient exceeded 0.7 for any pairwise combination. We included categorical fixed effects for migratory status (migratory or resident) and month (March, April, May, June, or July) because latent parasite infections are known to intensify at the commencement of the breeding season in early spring (Valkiunas 2004). We also included random effects for phylogenetic covariance and independent species variance because infection dynamics are influenced both by host–parasite coevolutionary history (Poulin 2010) and by species traits that mediate exposure (Medeiros et al. 2015). PGLMMs containing all predictors were fit to each of the four response variables derived from our analysis of avian blood samples: haemosporidian genus richness, presence of *Plasmodium*, presence of *Haemoproteus*, and presence of *Leucocytozoon*. Haemosporidian genus richness was quantified as an integer representing the number of parasite genera detected in a host and ranged from zero (no infection) to three (triple infection with *Plasmodium, Haemoproteus*, and *Leucocytozoon*). Richness was modeled using a PGLMM with a Poisson distribution and log link. The presence of *Plasmodium*, *Haemoproteus*, and *Leucocytozoon* was quantified as a binary variable and modeled using a PGLMM with a binomial distribution and logit link. For all models, we evaluated the significance of predictors using an alpha value of 0.05.

Finally, we used a generalized linear model (GLM) with a binomial distribution and logit link to evaluate whether (1) site contaminant history and (2) season of capture predicted the presence of *Plasmodium* in *Culex* vectors. Site contaminant history was included as a categorical fixed effect indicating whether a site was CCR, NFP, or reference. Because of a lower sample size of *Culex* compared to passerines, we evaluated “season” instead of “month” as a fixed effect. The variable “season” contained two categories: spring (April and May) and summer (June and July).

## 3 Results

We collected 334 blood samples from 329 individuals representing 31 bird species, including 13 resident species and 18 migratory species. Five individuals were sampled twice, with at least 1 month between sampling events. Because metal concentrations and parasite infections are dynamic in the blood throughout a single season, these five repeat samples were included as separate observations. The most common bird species were white‐eyed vireo (*Vireo griseus; n* = 47), Carolina wren ( *Thryothorus ludovicianus* ; *n* = 39), common yellowthroat (*Geothylpis trichas*; *n* = 39), northern cardinal ( *Cardinalis cardinalis* ; *n* = 37), and tufted titmouse ( *Baeolophus bicolor* ; *n* = 33). Other species sampled at all reference and contaminated site types include great‐crested flycatcher ( *Myiarchus crinitus* ; *n* = 18), red‐eyed vireo ( *Vireo olivaceus* ; *n* = 16), summer tanager ( *Piranga rubra* ; *n* = 12), Louisiana waterthrush ( *Parkesia motacilla* ; *n* = 10), pine warbler ( *Setophaga pinus* ; *n* = 10), common grackle ( *Quiscalus quiscula* ; *n* = 10), red‐winged blackbird ( *Agelaius phoeniceus* ; *n* = 6), and Acadian flycatcher ( *Empidonax virescens* ; *n* = 4), as well as the “near‐passerine” downy woodpecker ( *Dryobates pubescens* ; *n* = 9), which we included in our analysis. The capture dates, locations, and trace element concentrations for all individual birds are available as Supporting Information.

**FIGURE 1:**
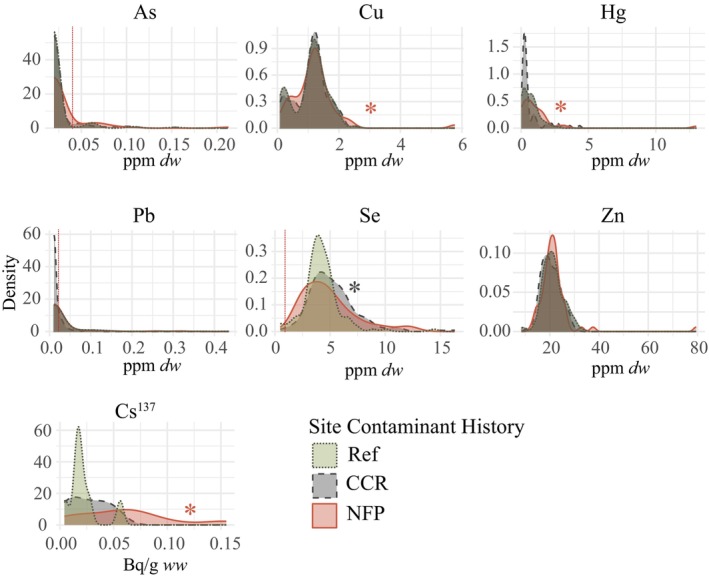
Density plots showing the distribution of As, Cu, Hg, Pb, Se, and Zn concentrations (ppm dry weight) in the blood, and ^137^Cs activity (Bq/g wet weight) in the whole body of passerine birds inhabiting areas affected by nuclear fission products (NFP) or coal combustion residuals (CCR) and reference areas (Ref) on the Savannah River Site. Red, vertical lines indicate method detection limits for ICP‐MS or DMA. The plot for ^137^Cs excludes non‐detects. Asterisks signify whether an element was significantly elevated at CCR or NFP sites compared to Ref sites, as indicated by PGLMMs (*α* = 0.05).

Zinc, Cu, and Hg were detected in all samples. Selenium was detected in 99% of samples (MDL = 0.96 ppm), Pb in 18% of samples (MDL = 0.02 ppm), As in 11% of samples (MDL = 0.04 ppm), and ^137^Cs in 21% of samples (MDA = 1.56 Bq). Average percent recoveries for the certified reference materials were within the accepted range of 80% to 120% for all elements. Blood Se was significantly higher among birds inhabiting CCR sites (*μ* = 5.3, SE = 0.2 ppm *dw*) compared to Ref sites (*μ* = 4.2, SE = 0.2 ppm *dw*; *β* = 1.0, *p* < 0.001), and whole‐body activity concentrations of Cs^137^ were higher among birds inhabiting NFP sites (*μ* = 0.02, SE = 0.00 Bq/g *ww*) compared to reference sites (*μ* = 0.00, SE = 0.00 Bq/g *ww*; *β* = 1.12, *p* = 0.02). Additionally, birds at NFP sites had higher blood Cu (*μ* = 1.2, SE = 0.1 ppm *dw*) and blood Hg (*μ* = 1.3, SE = 0.4 ppm *dw*) compared to birds at reference sites (Cu *μ* = 1.1, SE = 0.0 ppm *dw*; *β* = 0.2 ± 0.1, *p* = 0.001; Hg *μ* = 0.70, SE = 0.1 ppm *dw*; *β* = 0.6 ± 0.2, *p* = 0.001). The concentrations of other trace elements varied among individuals but were not significantly predicted by site contaminant history (Figure 1). Independent species variance was higher than phylogenetic covariance for all contaminants (Table S1). We observed evidence of phylogenetic covariation in concentrations of Zn (*σ* ^2^ = 0.4), As (*σ* ^2^ = 0.1), and Pb (*σ* ^2^ = 0.4), but not in concentrations of Se, Cs^137^, Cu, or Hg (all *σ* ^2^ < 0.001). Because Pb and As were detected in a small percentage of individuals and were not associated with the use of contaminated habitat, they were excluded from further analysis.

The prevalence of haemosporidian parasites in passerines captured on the Savannah River Site was 68.0%. *Plasmodium* infections were found in 55.1% of samples (*n* = 184), *Haemoproteus* in 29.3% of samples (*n* = 98), and *Leucocytozoon* in 12.9% of samples (*n* = 43). We observed coinfections in 26.3% of samples (*n* = 88), including 3.3% (*n* = 10) with triple infections.

We did not observe collinearity between predictors. Trace element concentrations within birds were weakly correlated, with the strongest correlations between Se and Hg (*r* = 0.38), followed by Cu and Zn (*r* = 0.31). Because of limitations on blood sample quantity and handling time for the smallest birds, sample sizes were lower for blood Hg (*n* = 184) and ^137^Cs activity (*n* = 158) than for the other trace elements. Consequently, we evaluated the effects of Hg and ^137^Cs on parasite infection separately from the other contaminants, and to resolve model singularity, we used a simplified random effects structure that excluded phylogenetic covariance.

**FIGURE 2:**
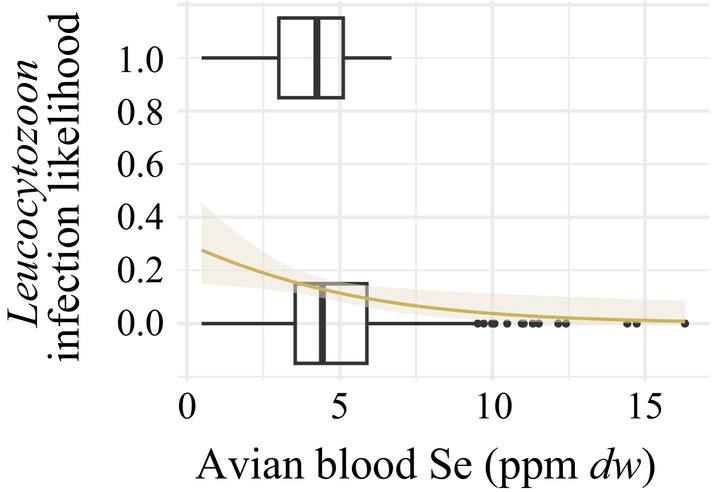
Average predicted likelihood of *Leucocytozoon* infection across the range of blood Se concentrations observed in passerines on the Savannah River Site, with standard error ribbons. Predicted values were generated from the fitted binomial PGLMM with logit link (Table S2) using *phyr::Pglmm_predicted_values* (Li et al. 2020) and were back‐transformed to the probability scale. Boxplots represent the observed blood Se concentrations in birds with and without *Leucocytozoon* infection. *Leucocytozoon* was not found in birds with blood Se concentrations > 6.7 ppm *dw*.

A higher concentration of Se in the blood was associated with a lower likelihood of *Leucocytozoon* infection (*β* = −0.63, SE = 0.30, *p* = 0.03; Table S2). The presence of *Leucocytozoon* adhered to an apparent blood Se threshold of 6.7 ppm *dw*, where birds with blood Se values above this threshold were uninfected (Figure 2). Blood concentrations of Zn, Cu, or Hg and whole‐body activity concentrations of ^137^Cs were not associated with an altered likelihood of infection by any of the haemosporidian genera (Table S2). Blood contaminant concentrations and whole‐body activity concentrations were not associated with altered haemosporidian genus richness.

Compared to birds captured in July, birds captured in April harbored a greater number of coinfections (*β* = 0.54, SE = 0.21, *p* = 0.008) and were significantly more likely to be infected with *Plasmodium* (*β* = 1.08, SE = 0.48, *p* = 0.03), *Haemoproteus* (*β* = 1.34, SE = 0.66, *p* = 0.04), and *Leucocytozoon* (*β* = 2.37, SE = 0.85, *p* = 0.005). Birds captured in May were also more likely to be infected with *Haemoproteus* (*β* = 1.40, SE = 0.64, *p* = 0.03) and *Leucocytozoon* (*β* = 1.83, SE = 0.87, *p* = 0.03). Migratory status was not a significant predictor of parasitism in any model.

**FIGURE 3:**
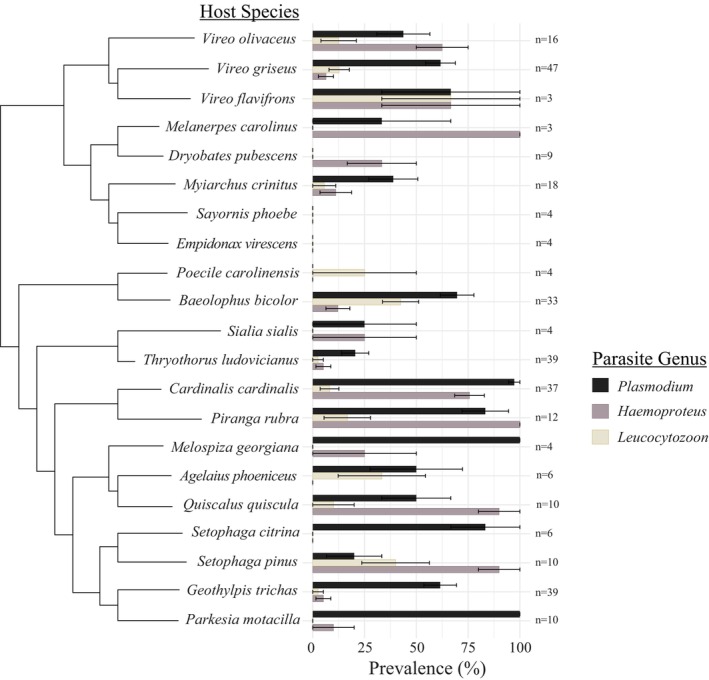
Phylogenetic tree topology of passerine host species on the Savannah River Site, excluding species for which *n* < 3, and prevalences of haemosporidian parasite genera. Error bars represent standard error.

The likelihood of infection with each of the three haemosporidian parasite genera differed by host species (Figure 3). We observed the highest phylogenetic covariation in the likelihood of *Plasmodium* infection across avian host species (*σ* ^2^ = 0.69), followed by haemosporidian genus richness (*σ* ^2^ = 0.13). We found no evidence of phylogenetic covariation in the likelihood of infection with *Haemoproteus* or *Leucocytozoon* (*σ* ^2^ < 0.001). Conversely, independent species variance in likelihood of infection was higher for *Haemoproteus* (*σ* ^2^ = 3.7) and *Leucocytozoon* (*σ* ^2^ = 1.2) than for *Plasmodium* (*σ* ^2^ = 0.7) and haemosporidian genus richness (*σ* ^2^ = 0.1).

**FIGURE 4:**
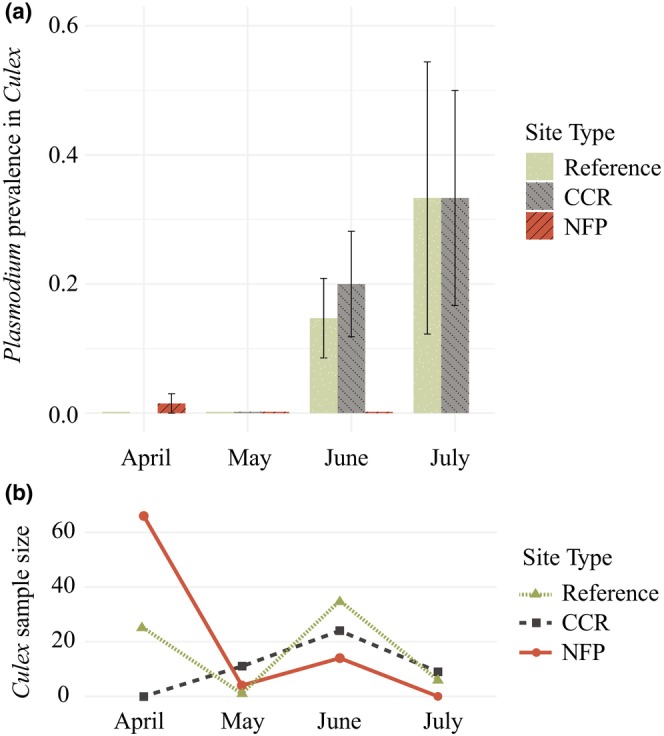
(a) Prevalence (±SE) of *Plasmodium* in *Culex* mosquitoes sampled from April through July at sites affected by coal combustion residuals or nuclear fission products and reference sites. Prevalence is expressed as the proportion of infected individuals in each sample. (b) Number of *Culex* captured per sampling event.

We captured 195 *Culex* vectors, including 65 individuals at reference sites, 45 at CCR sites, and 85 at NFP sites. Capture rates varied widely between months (Figure 4). The overall prevalence of *Plasmodium* in *Culex* was 8.2%. *Culex* vectors captured in June and July were significantly more likely to be infected with *Plasmodium* compared to those captured in April and May (*β* = 2.46, SE = 1.09, *p* = 0.02; Table S3). There was no significant effect of site contaminant history on *Plasmodium* infection in *Culex* mosquitoes.

## 4 Discussion

This study investigated whether waste‐derived trace elements altered *Plasmodium*, *Haemoproteus*, and *Leucocytozoon* infection in communities of passerine birds and *Culex* vectors. Passerines inhabiting CCR sites accumulated excess concentrations of Se in their blood compared to those at reference sites, which aligns with prior evidence that Se derived from CCR is bioavailable and transferred to terrestrial wildlife (Bryan et al. 2003; Meyer et al. 2014; Werner et al. 2025). We found that higher concentrations of Se in the blood were associated with a lower likelihood of *Leucocytozoon* infection within individuals. This finding contradicts our first hypothesis and provides evidence for the alternative—that sublethal Se exposure decreases parasitism within individuals by fostering unfavorable conditions for some parasites. Neither blood Se nor *Leucocytozoon* infection was predicted by phylogenetic relatedness among hosts, but both varied among species, suggesting that Se uptake and associated changes in parasitism are mediated by variation in contaminant exposure among species with different habitat use strategies.

Selenium is required in trace amounts by living organisms and plays an essential role in immune function (Hoffman and Berry 2008). As a component of the antioxidant glutathione peroxidase, Se enables hosts to cope with the inflammatory response and oxidative damage caused by parasite infection (Cantor and Tarino 1982; Nelson et al. 2016). Experimental studies have demonstrated that dietary Se supplementation increases glutathione peroxidase activity and reduces the severity of disease in protozoan‐infected animal models (Cantor and Tarino 1982; Huang and Yang 2002), and treatment with selenium nanoparticles produces morphological deformities in some parasites (Arafa et al. 2023; Nagdy et al. 2025). On the SRS, birds receiving higher doses of Se because of trophic contaminant transfer may be more capable of amassing an immune response that clears *Leucocytozoon* infection. We did not observe a comparable relationship between blood Se concentration and the presence of *Haemoproteus* or *Plasmodium*, possibly because of the chronic nature of infection with these parasites in passerine hosts. In a long‐term field study, Rooyen et al. (2013) suggested that the passerine immune system may be better equipped to defend against *Leucocytozoon* compared to *Plasmodium* and *Haemoproteus* after observing that *Leucocytozoon* in great tits ( *Parus major* ) had higher turnover across seasons than the other genera.

The factors governing haemosporidian co‐infections in passerines are still poorly understood. We hypothesized that contaminant exposure would weaken host immune response and allow multiple haemosporidia genera to establish within hosts. Contrary to our predictions, trace element burdens were not associated with greater haemosporidian parasite richness in passerines. We did find strong evidence that haemosporidian richness and the likelihoods of single infection with *Plasmodium*, *Haemoproteus*, and *Leucocytozoon* were highest in the months of April and May, which coincide with peak breeding season in our population. This reflects a well‐studied temporal cycle of avian haemosporidian infection where, during breeding, passerines invest maximal energy in reproduction and less energy in immune function (Astudillo et al. 2013; Knowles et al. 2011). The reduced strength of immune defenses facilitates the relapse of latent parasite infections and allows a greater number of parasite genera to compete for limited resources within the host (Valkiunas 2004). Phylogenetic relatedness also predicted haemosporidian parasite richness, suggesting that a species' coevolutionary history with multiple haemosporidian genera, combined with temporal cycles in immune function, is a stronger driver of co‐infection susceptibility than exposure to the metals, metalloids, and radionuclides derived from waste on the SRS.

A remaining unknown in our system is whether contaminant exposure and/or parasite infection affects passerine breeding success. Stressor‐induced releases from parasitism will not benefit the fitness of hosts if they are outweighed by reductions in survival or reproductive output, and therefore an understanding of the implications of Se accumulation on passerine populations requires longer‐term assessments of these endpoints. In a population of wild common eiders ( *Somateria mollissima* ) exposed to Pb accumulation, Morrill et al. (2019) found that infection with intestinal helminths was beneficial to host survival, likely because of Pb sequestration by the parasites. However, elevated blood Pb negatively affected *S. mollissima* clutch size regardless of the presence of intestinal parasites. Breeding birds are known to transfer excess blood Se to their eggs, leading to reduced hatchability at high enough levels (Bryan et al. 2003). The average Se concentrations observed in birds at our CCR sites exceed blood thresholds of concern for reproductive impairment in several species (4.8 ppm *dw*; Ohlendorf and Heinz 2011; Werner et al. 2025). The possibility that excess trace elements can decrease parasitism while also lowering breeding success suggests a complex fitness landscape in contaminated habitats and presents an intriguing direction for future study.

We did not find evidence in support of our second hypothesis that parasite transmission is disrupted among vectors inhabiting contaminated sites. The likelihood of *Plasmodium* infection in *Culex* vectors did not differ between waste and reference sites, but *Culex* were more likely to be infected in June and July compared to April and May. This reflects the expected pattern of the annual infection cycle, where the avian breeding season triggers a shift from latent to acute *Plasmodium* infection in birds, which in turn infect emergent *Culex* females seeking a bloodmeal (Valkiunas 2004). The lack of a site‐level difference in *Plasmodium* infection among *Culex* suggests that environmental contamination might not hinder the transmission of *Plasmodium* from hosts to vectors by negatively impacting the latter's fitness in our system. We did not sample throughout the entire *Culex* breeding season and therefore cannot determine whether the observed temporal increases in *Culex* infection prevalence were followed by a second peak in *Plasmodium* prevalence among hosts. However, the lack of difference in *Plasmodium* infection likelihood between year‐round resident birds and migratory birds suggests that transmission of *Plasmodium* from vectors to avian hosts is not disrupted on the SRS landscape. Our findings contradict prior laboratory experiments that have demonstrated reduced survival and vector competency among mosquitos exposed to heavy metals (Barreaux et al. 2016; Neff and Dharmarajan 2021) and ionizing radiation (Cunningham et al. 2020), though these experiments were conducted on naïve mosquitos rather than individuals collected from polluted environments. Other studies have demonstrated increased metallothionein expression and associated trace element tolerance in *Culex* and *Anopheles* exposed to heavy metals over multiple generations (Mireji et al. 2010; Sarkar et al. 2004). Given that environmental contaminant concentrations are spatially heterogenous at our sites (Fletcher et al. 2019; SRNS 2022), *Culex* populations could also be sustained by patches of minimally polluted breeding habitat. Alternatively, our variable *Culex* capture rates and low observed *Plasmodium* prevalence could have limited our power to detect differences in *Culex* infection across sites. The low prevalence observed in our sample could be driven, in part, by the presence of *Culex territans* , an amphibian feeder that does not vector avian *Plasmodium* (Reinhold et al. 2023). The effects of trace element contamination on mosquito vectors warrant further investigation and would benefit from field studies that employ a greater number of site replicates, higher trapping frequency, longer‐term sampling periods, and species‐level identification.

Our findings highlight counterintuitive effects of trace element contaminants on host–parasite ecology and raise important questions. First, the association between increased dietary Se and decreased haemosporidian infection in passerines should be investigated further. Experimental manipulation of dietary Se, along with regular measurements of the presence and parasitemia of specific haemosporidian lineages and of host oxidative stress biomarkers, could reveal causative relationships between Se supplementation and finer‐scale metrics of disease severity in wild passerines. Cornet et al. (2014) conducted a similar study and found that dietary supplementation led to lower *Plasmodium relictum* parasitemia in domestic canaries (*Serinus canaria*); yet further work is required to identify the roles of specific dietary trace elements in this response. Micronutrient supplementation could have practical implications for improving the resistance of wildlife threatened by disease, although such a strategy could increase host reservoir capacity and would warrant long‐term population monitoring. Second, our findings provide a limited snapshot of altered vector‐borne parasite dynamics on a contaminated landscape at the scale of a single season. The bioavailability of contaminants, the susceptibility of hosts to infection, and the rate of exposure to vectors vary across seasonal, annual, and multi‐year time scales and depend on a multitude of individual and environmental variables (Hawley and Altizer 2011; Noyes et al. 2009; Stephens et al. 2016). Further studies should investigate how waste influx influences patterns of wildlife parasitism over greater spatial and temporal scales, ideally by quantifying long‐term trends in infection intensity among individuals with varying use of contaminated habitat. Trace elements derived from CCR and other waste products circulate widely among wildlife and have implications for their parasites, including those that pose zoonotic risks to humans. Although a comparatively large body of literature has examined the nuanced dynamics of parasite transmission at the urban‐wildland interface, fewer studies have been conducted in habitats that remain structurally intact but are chemically altered by anthropogenic contaminants. Such environments attract a variety of wildlife and represent important areas for future research.

## Author Contributions

**Courtney S. Werner:** conceptualization (equal), data curation (lead), formal analysis (lead), funding acquisition (equal), investigation (equal), visualization (lead), writing – original draft (lead), writing – review and editing (lead). **Mary Chapman:** conceptualization (equal), data curation (supporting), formal analysis (equal), investigation (equal), methodology (equal), project administration (equal), validation (equal), writing – original draft (supporting), writing – review and editing (supporting). **Daniel A. H. Peach:** conceptualization (equal), funding acquisition (equal), investigation (equal), methodology (equal), project administration (supporting), resources (equal), supervision (supporting), validation (equal), writing – review and editing (equal). **Travis L. DeVault:** conceptualization (equal), funding acquisition (equal), investigation (equal), methodology (equal), project administration (equal), resources (equal), supervision (equal), validation (equal), writing – review and editing (equal). **Olin E. Rhodes Jr.:** conceptualization (equal), funding acquisition (equal), investigation (equal), methodology (equal), project administration (equal), resources (equal), supervision (lead), validation (equal), writing – review and editing (equal).

## Funding

This material is based upon work supported by the U.S. Department of Energy Office of Environmental Management and National Nuclear Security Administration under Award Number DE‐EM0005228 to the University of Georgia Research Foundation and by the National Science Foundation Graduate Research Fellowship under Award Number 2236869.

## Ethics Statement

All collections were completed under a Federal Migratory Bird Scientific Collection Permit (#MB65214A), a South Carolina Wildlife Collection Permit (#SC‐08‐2022), and a University of Georgia Animal Care and Use Protocol (#A2021 05‐003‐Y3‐A8).

## Conflicts of Interest

The authors declare no conflicts of interest.

## Supporting information


**Data S1:** ece372681‐sup‐0001‐DataS1.xlsx.

**Data S2:** ece372681‐sup‐0002‐DataS2.xlsx.

**Appendix S1:** ece372681‐sup‐0003‐AppendixS1.docx.

## Data Availability

All required data are uploaded as Supporting Information.
