# Supplementary material for: Use of Contaminated Habitat and Associated Selenium Uptake Mediate Haemosporidian Parasite Infections in Wild Passerine Birds
Source: Ecol Evol. 2026 Jan 13;16(1):e72681. doi: 10.1002/ece3.72681 (PMC12796830; doi:10.1002/ece3.72681)
Supplement: Supplementary file 3 — Appendix S1: ece372681‐sup‐0003‐AppendixS1.docx. [file ECE3-16-e72681-s003.docx]

**Supplementary Table 1**. Phylogenetic generalized linear mixed effects models showing predictors of contaminant concentrations in passerines on the Savannah River Site. Predictors marked with asterisks were considered significant based on an alpha value of 0.05.

| Selenium ~ site contaminant history + (1\|species), family = gaussian(link = “identity”) | | | | |
| --- | --- | --- | --- | --- |
| **Fixed Effect** | **Coefficient** | **Std. Err.** | **z value** | **p value** |
| Intercept | 4.32 | 0.35 | 12.35 | <0.001 |
| CCR site* | 1.04 | 0.21 | 4.89 | <0.001 |
| NFP site | 0.35 | 0.27 | 1.31 | 0.19 |
| **Random Effect** | **Variance** | **Std. Dev.** |  |  |
| Species – identity | 2.61 | 1.62 |  |  |
| Species – phylogeny | <0.001 | 1.66 |  |  |
|  |  |  |  |  |
| Zinc ~ site contaminant history + (1\|species), family = gaussian(link = “identity”) | | | | |
| **Fixed Effect** | **Coefficient** | **Std. Err.** | **z value** | **p value** |
| Intercept | 20.31 | 0.73 | 27.71 | <0.001 |
| CCR site | -0.65 | 0.59 | -1.11 | 0.27 |
| NFP site | 1.24 | 0.75 | 1.66 | 0.10 |
| **Random Effect** | **Variance** | **Std. Dev.** |  |  |
| Species – identity | 3.16 | 1.78 |  |  |
| Species – phylogeny | 0.42 | 0.65 |  |  |
|  |  |  |  |  |
| Copper ~ site contaminant history + (1\|species), family = gaussian(link = “identity”) | | | | |
| **Fixed Effect** | **Coefficient** | **Std. Err.** | **z value** | **p value** |
| Intercept | 1.12 | 0.08 | 14.63 | <0.001 |
| CCR Site | 0.02 | 0.05 | 0.51 | 0.61 |
| NFP Site* | 0.19 | 0.06 | 3.21 | 0.001 |
| **Random Effect** | **Variance** | **Std. Dev.** |  |  |
| Species – identity | 0.12 | 0.35 |  |  |
| Species – phylogeny | <0.001 | <0.001 |  |  |
|  |  |  |  |  |
| Arsenic ~ site contaminant history + (1\|species), family = binomial(link = logit) | | | | |
| **Fixed Effect** | **Coefficient** | **Std. Err.** | **z value** | **p value** |
| Intercept | -2.08 | 0.43 | -4.90 | <0.001 |
| CCR site | 0.28 | 0.42 | 0.67 | 0.50 |
| NFP Site | 0.59 | 0.48 | 1.21 | 0.23 |
| **Random Effect** | **Variance** | **Std. Dev.** |  |  |
| Species – identity | 0.27 | 0.52 |  |  |
| Species – phylogeny | 0.14 | 0.38 |  |  |
|  |  |  |  |  |
| Lead ~ site contaminant history + (1\|species), family = binomial(link = logit) | | | | |
| **Fixed Effect** | **Coefficient** | **Std. Err.** | **z value** | **p value** |
| Intercept | -1.09 | 0.56 | -1.97 | 0.05 |
| CCR site | 0.26 | 0.39 | 0.68 | 0.49 |
| NFP site | -0.56 | 0.54 | -1.03 | 0.30 |
| **Random Effect** | **Variance** | **Std. Dev.** |  |  |
| Species – identity | 1.87 | 1.36 |  |  |
| Species – phylogeny | 0.35 | 0.59 |  |  |
|  |  |  |  |  |
| Mercury ~ site contaminant history + (1\|species), family = gaussian(link = identity) | | | | |
| **Fixed Effect** | **Coefficient** | **Std. Err.** | **z value** | **p value** |
| Intercept | 0.65 | 0.20 | 3.35 | <0.001 |
| CCR site | 0.07 | 0.15 | 0.44 | 0.66 |
| NFP site* | 0.59 | 0.19 | 3.19 | 0.001 |
| **Random Effect** | **Variance** | **Std. Dev.** |  |  |
| Species – identity | 0.65 | 0.81 |  |  |
| Species – phylogeny | <0.001 | 0.001 |  |  |
|  |  |  |  |  |
| Cesium-137 ~ site contaminant history + (1\|species), family = binomial(link = logit) | | | | |
| **Fixed Effect** | **Coefficient** | **Std. Err.** | **z value** | **p value** |
| Intercept | -1.99 | 0.44 | -4.51 | <0.001 |
| CCR site | 0.90 | 0.62 | 1.46 | 0.14 |
| NFP site* | 1.12 | 0.50 | 2.25 | 0.02 |
| **Random Effect** | **Variance** | **Std. Dev.** |  |  |
| Species – identity | 0.83 | 0.91 |  |  |
| Species – phylogeny | <0.001 | <0.001 |  |  |
|  |  |  |  |  |

| **Supplementary Table 2.** Phylogenetic generalized linear mixed effects models showing predictors of haemosporodian parasite infection in passerines on the Savannah River Site. Months are compared to reference level “July”. Predictors marked with asterisks were considered significant based on an alpha value of 0.05. | | | | |
| --- | --- | --- | --- | --- |
| Avian parasite richness ~ Cu + Zn + Se + month + (1\|species), family = Poisson(link = “log”) | | | | |
| **Fixed Effect** | **Coefficient** | **Std. Err.** | **z value** | **p value** |
| Intercept | -0.25 | 0.30 | -0.83 | 0.41 |
| Cu | 0.08 | 0.11 | 0.75 | 0.46 |
| Zn | -0.12 | 0.09 | -1.38 | 0.17 |
| Se | 0.02 | 0.07 | 0.24 | 0.81 |
| March | 0.09 | 0.23 | 0.38 | 0.70 |
| April* | 0.54 | 0.21 | 2.65 | 0.008 |
| May | 0.29 | 0.21 | 1.41 | 0.16 |
| June | 0.12 | 0.21 | 0.54 | 0.59 |
| migratory | -0.03 | 0.23 | -0.15 | 0.88 |
| **Random Effect** | **Variance** | **Std. Dev** |  |  |
| Species – identity | <0.001 | 0.002 |  |  |
| Species – phylogeny | 0.13 | 0.35 |  |  |
|  |  |  |  |  |
| Avian *Plasmodium*  ~ Cu + Zn + Se + month + (1\|species), family = binomial(link= “logit”) | | | | |
| **Fixed Effect** | **Coefficient** | **Std. Err.** | **z value** | **p value** |
| Intercept | 0.44 | 0.74 | 0.60 | 0.55 |
| Cu | 0.02 | 0.25 | 0.09 | 0.93 |
| Zn | -0.27 | 0.19 | -1.44 | 0.15 |
| Se | 0.32 | 0.19 | 1.74 | 0.08 |
| March | -0.05 | 0.47 | -0.10 | 0.92 |
| April* | 1.08 | 0.48 | 2.23 | 0.03 |
| May | -0.37 | 0.46 | -0.80 | 0.43 |
| June | 0.03 | 0.42 | 0.08 | 0.94 |
| migratory |  |  |  |  |
| **Random Effect** | **Variance** | **Std. Dev** |  |  |
| Species – identity | 0.38 | 0.62 |  |  |
| Species – phylogeny | 0.69 | 0.83 |  |  |
|  |  |  |  |  |
|  |  |  |  |  |
| Avian *Haemoproteus*  ~ Cu + Zn + Se + month + (1\|species), family = binomial(link= “logit”) | | | | |
| **Fixed Effect** | **Coefficient** | **Std. Err.** | **z value** | **p value** |
| Intercept | -1.26 | 0.83 | -1.51 | 0.13 |
| Cu | 0.21 | 0.35 | 0.61 | 0.54 |
| Zn | -0.16 | 0.28 | -0.56 | 0.58 |
| Se | 0.20 | 0.24 | 0.82 | 0.41 |
| March | -0.35 | 0.72 | -0.50 | 0.62 |
| April* | 1.34 | 0.66 | 2.03 | 0.04 |
| May* | 1.40 | 0.64 | 2.19 | 0.03 |
| June | 0.64 | 0.67 | 0.96 | 0.34 |
| migratory | -0.68 | 0.89 | -0.76 | 0.45 |
| **Random Effect** | **Variance** | **Std. Dev** |  |  |
| Species – identity | 3.67 | 1.92 |  |  |
| Species – phylogeny | <0.001 | <0.001 |  |  |
|  |  |  |  |  |
| Avian *Leucocytozoon*  ~ Cu + Zn + Se + month + (1\|species), family = binomial(link= “logit”) | | | | |
| **Fixed Effect** | **Coefficient** | **Std. Err.** | **z value** | **p value** |
| Intercept | -3.90 | 0.90 | -4.33 | 0.00 |
| Cu | 0.11 | 0.34 | 0.32 | 0.75 |
| Zn | -0.40 | 0.31 | -1.32 | 0.19 |
| Se* | -0.63 | 0.30 | -2.13 | 0.03 |
| March | 1.37 | 0.88 | 1.56 | 0.12 |
| April* | 2.37 | 0.85 | 2.78 | 0.005 |
| May* | 1.83 | 0.87 | 2.11 | 0.03 |
| June | 0.56 | 0.93 | 0.61 | 0.54 |
| migratory |  |  |  |  |
| **Random Effect** | **Variance** | **Std. Dev** |  |  |
| Species – identity | 1.15 | 1.07 |  |  |
| Species – phylogeny | <0.001 | <0.001 |  |  |
|  |  |  |  |  |

**Supplementary Table 3.** Generalized linear model showing predictors of *Plasmodium* infection in *Culex* sp. on the Savannah River Site. Season “June/July” is compared to reference level “April/May” Predictors marked with asterisks were considered significant based on an alpha value of 0.05.

| *Plasmodium* ~ site contaminant history + season + (1\|species), family = binomial(link = “logit”) | | | | | | | |
| --- | --- | --- | --- | --- | --- | --- | --- |
| **Fixed Effect** | | **Coefficient** | | **Std. Err.** | | **z value** | **p value** |
| Intercept | | -4.08 | | 1.09 | | -3.73 | <0.001 |
| June/July* | | 2.46 | | 1.09 | | 2.26 | 0.02 |
| CCR site | | 0.40 | | 0.57 | | 0.70 | 0.49 |
| NFP site | | -1.34 | | 1.13 | | -1.18 | 0.24 |
|  |  | |  | |  |  |  |
|  | |  | |  | |  |  |
